# A shared synapse architecture for efficient FPGA implementation of autoencoders

**DOI:** 10.1371/journal.pone.0194049

**Published:** 2018-03-15

**Authors:** Akihiro Suzuki, Takashi Morie, Hakaru Tamukoh

**Affiliations:** Graduate School of Life Science and Systems Engineering, Kyushu Institute of Technology, 2-4 Hibikino, Wakamatsu-ku, Kitakyushu 808-0196, Japan; Lanzhou University of Technology, CHINA

## Abstract

This paper proposes a shared synapse architecture for autoencoders (AEs), and implements an AE with the proposed architecture as a digital circuit on a field-programmable gate array (FPGA). In the proposed architecture, the values of the synapse weights are shared between the synapses of an input and a hidden layer, and between the synapses of a hidden and an output layer. This architecture utilizes less of the limited resources of an FPGA than an architecture which does not share the synapse weights, and reduces the amount of synapse modules used by half. For the proposed circuit to be implemented into various types of AEs, it utilizes three kinds of parameters; one to change the number of layers’ units, one to change the bit width of an internal value, and a learning rate. By altering a network configuration using these parameters, the proposed architecture can be used to construct a stacked AE. The proposed circuits are logically synthesized, and the number of their resources is determined. Our experimental results show that single and stacked AE circuits utilizing the proposed shared synapse architecture operate as regular AEs and as regular stacked AEs. The scalability of the proposed circuit and the relationship between the bit widths and the learning results are also determined. The clock cycles of the proposed circuits are formulated, and this formula is used to estimate the theoretical performance of the circuit when the circuit is used to construct arbitrary networks.

## Introduction

Deep neural networks (DNNs) are known for their high levels of performance in machine learning applications [1]. DNNs are general models of deeply stacked neural networks and include multi-layer perceptrons (MLPs) and convolutional neural networks (CNNs), restricted Boltzmann machines (RBMs) and autoencoders (AEs).

MLPs are the most basic DNNs model and they are applied to classification tasks [2]. CNNs are modeled on the structure of the human visual cortex and mainly focus on image recognition tasks [3], while RBMs are generative models that can learn a probability distribution over an input dataset [1, 4]. AEs obtain a representation for an input dataset and have several versions [5–8]. AEs are used in a wide variety of different applications; low-light image enhancement [9] and dimensionality reduction for image processing [10] in image processing domain; the hash function for data mining [11], tomographic reconstruction from dynamic positron emission tomography [12], and the extractor for latent representations of documents [13].

In most DNNs, processes are divided into pre-training and fine-tuning phases [14]. RBMs and AEs are stacked so as to be used in the pre-training phase [15, 16]. In this phase, the objectives are to train a part of an entire network and obtain effective initial parameters for the fine-tuning via unsupervised learning. In the fine-tuning phase, the pre-trained network is used as a feature extractor so that the entire network, including a classifier, can be tuned via supervised learning. In both phases, a great amount of data and computational resources are required for learning.

It is expected that in the future, DNNs will be loaded onto embedded systems such as mobile devices, robots, and automobiles [17–19]. Because embedded systems with DNNs are expected to be used in everyday items, the embedded systems should have the following features: real-time processing, low power consumption, and situational-responsive capabilities. With all of these considerations in mind, hardware is considered to be a better choice than software for these systems. The pieces of hardware often used for the implementation of DNNs are graphics processing units (GPUs), dedicated very large-scale integration (VLSI) chips, and field-programmable gate arrays (FPGAs).

Several libraries and frameworks have been developed for the implementation of DNNs via GPUs; these include Theano (which is a Python library) [20] and Caffe (a deep learning framework) [21], Tensor Flow and Chianer (Python-based deep learning frameworks) [22, 23]. Some DNNs, such as some MLPs [24, 25], RBMs [26, 27], and CNNs [28–31], have been developed as dedicated chips. One of the report uses RBMs for training and AEs for inference [32]. In addition to these examples of the hardware implementations of DNNs, FPGA implementations of CNNs and RBMs have also been reported in [33–38]. Unlike other hardware implementations, FPGAs are more suitable for implementing DNNs onto embedded systems, as FPGAs require less power than GPUs and, unlike dedicated chips, their internal circuits can be reconfigured by users.

A few reports have also been published about the implementation of AE into FPGAs [39, 40]. One of these proposed a digital circuit of AEs by high-level synthesis (HLS) [39]. In that particular study, the circuit generated by HLS was loaded onto an Altera Stratix V GS D5 FPGA. However, a comparison between regular GPUs, mobile GPUs, and FPGAs found that the HLS-designed circuit had several drawbacks. Because HLS automatically generates a circuit architecture from an algorithmic description, the generated circuit cannot effectively utilize FPGAs. The other relevant study to discuss here created a behavior model simulation for AEs [40]; in the simulation, a sparse AE was implemented and used for an image recognition task on a set of Kyoto pictures. Because the circuit used in the simulation required too many resources from the FPGA, it was impossible for the circuit to be implemented onto an actual FPGA. As stated above, the study of FPGA implementation for AEs has been still insufficient. The implementation of register transfer level (RTL) description is required to utilize the FPGA effectively, and small-but-adjustable architectures for AEs that can be implemented on actual FPGAs are also required.

This paper proposes a novel hardware architecture for AEs called a shared synapse architecture, and it demonstrates the results of implementing such AEs onto a digital circuit. This paper is an extended version of work published in [41]. The sharing synapse was originally reported in an earlier study [5], and another earlier study [42] called the method of sharing synapses as a tied weight technique; this technique restricts AEs so as to improve the performance of AEs’ reconstruction. We use a tied weight technique to efficiently use the resources of the FPGAs in our study. By assuming that the reconstruction of an AE is divided into those in the neuron and synapse modules, the shared synapse architecture is able to halve the number of the synapse modules.

In this paper, a digital circuit with the shared synapse architecture is implemented via a RTL description. The circuit is designed in an object-oriented manner and implemented as a basic model that could be scaled. In our experiments, we investigate the performance and scalability of the proposed digital circuit. This paper is organized as follows: Section 2 explains the basic algorithms of the AEs used along with the algorithm related to the proposed idea; in Section 3, we propose and describe in detail a novel hardware architecture for AEs; Section 4 looks at the implementation of the proposed AEs on a digital circuit via software experiments; Section 5 shows the results of the logic synthesis and verifies the circuit operation as AEs; in Section 6, the scalable functions of the proposed design circuit are investigated; Section 7 compares the present work with other related works; and in Section 8, we put forward our conclusions.

## Autoencoders

### Learning algorithm of autoencoders

**Fig 1:**
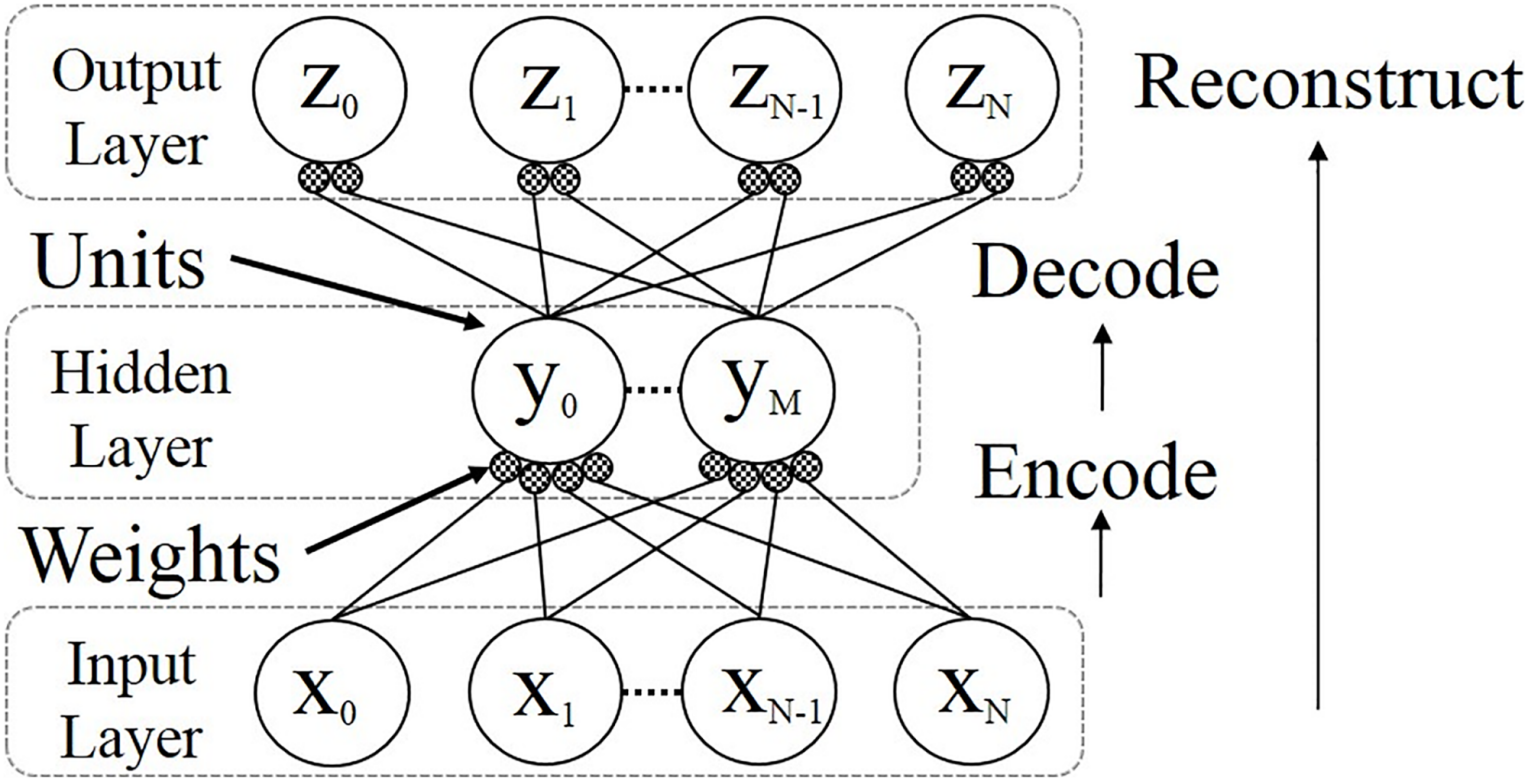
Autoencoder. Reprinted from [41] under a CC BY license, with permission from Springer Nature, original copyright 2016 (S1 File).

As shown in Fig 1, AEs are composed of three layers: an input, a hidden, and an output layer. Each layer has neurons that are connected to each other via synapses between the layers. The data of the neurons are handed over to the next layer via synapses, and each synapse has a weight value representing the transmission efficiency. In the hidden and output layers, each neuron has a bias that controls the firing probability.

The difference between the input and output values is measured by the cross-entropy error. This error is minimized by the gradient descent method, and the values of the weights and the biases are updated so that this error is reduced. These processes are repeated until the output values become to reconstruct input values.

If an input vector is assumed to be $\vec{x}$, a hidden one $\vec{y}$, and an output one $\vec{z}$, then the reconstruction and update flow can be represented by the sequence of equations shown below. In this flow, the hidden weight and bias vectors are $\vec{W}$ and $\vec{b}$, respectively, while the output ones are $\vec{W^{\prime }}$ and $\vec{b^{\prime }}$, respectively. The function *σ* is an activation function such as a sigmoidal function, and *η* is the learning rate. It should be noted that 1 is a vector of all ones, ⋅ is an inner product, and ∘ is the Hadamard product. The reconstruction and update flow can be represented as follows:

1. $\vec{x}$ is encoded and transformed in the hidden layer thereby becoming $\vec{y}$:
  $$\begin{array}{c} {\vec{y}}_{m}=\sigma \left(\sum_{n=0}^{N}{\vec{W}}_{mn}\cdot {\vec{x}}_{n}+{\vec{b}}_{m}\right)\;\;m=0,1\ldots ,M-1,M\;. \end{array}$$ (1)
2. $\vec{y}$ is decoded and transformed in the output layer, thereby becoming $\vec{z}$:
  $$\begin{array}{c} {\vec{z}}_{n}=\sigma \left(\sum_{m=0}^{M}{\vec{W^{\prime }}}_{nm}\cdot {\vec{y}}_{m}+{\vec{b^{\prime }}}_{n}\right)\;\;n=0,1\ldots ,N-1,N\;. \end{array}$$ (2)
3. The cross entropy, C, is measured using $\vec{x}$ and $\vec{z}$:
  $$\begin{array}{c} C\left(\vec{x},\vec{z}\right)=-\vec{x}\log\vec{z}-\left(1-\vec{x}\right)\log\left(1-\vec{z}\right) \end{array}$$ (3)
4. The update values for each parameters are obtained from the cross entropy error.
  $$\begin{array}{l} \Delta \vec{W}=\left(\vec{W}\cdot \left(\vec{x}-\vec{z}\right)\circ \vec{y}\circ \left(1-\vec{y}\right)\cdot {\vec{x}}^{T}\right)\\ +\left({\left(\left(\vec{x}-\vec{z}\right)\cdot {\vec{y}}^{T}\right)}^{T}\right) \end{array}$$ (4)
  $$\begin{array}{c} \Delta \vec{b}=(\vec{W}\cdot (\vec{x}-\vec{z})\circ \vec{y}\circ (1-\vec{y})) \end{array}$$ (5)
  $$\begin{array}{c} \Delta \vec{b^{\prime }}=\left(\vec{x}-\vec{z}\right) \end{array}$$ (6)
5. Each update value is added to the old parameters, which allows the new parameters to be obtained.
  $$\begin{array}{c} {\vec{W}}^{new}={\vec{W}}^{old}+\eta \Delta \vec{W} \end{array}$$ (7)
  $$\begin{array}{c} {\vec{b^{\prime }}}^{new}={\vec{b^{\prime }}}^{old}+\eta \Delta \vec{b^{\prime }} \end{array}$$ (8)
  $$\begin{array}{c} \vec{b^{new}}={\vec{b}}^{old}+\eta \Delta \vec{b^{\prime }} \end{array}$$ (9)

In this paper, these equations are computed on digital circuits; Eqs (1) and (2) are computed in a reconstruction module, Eqs (4), (5) and (6) at an update function module, and Eqs (7), (8) and (9) at an update execution module.

### Stacked autoencoders

**Fig 2:**
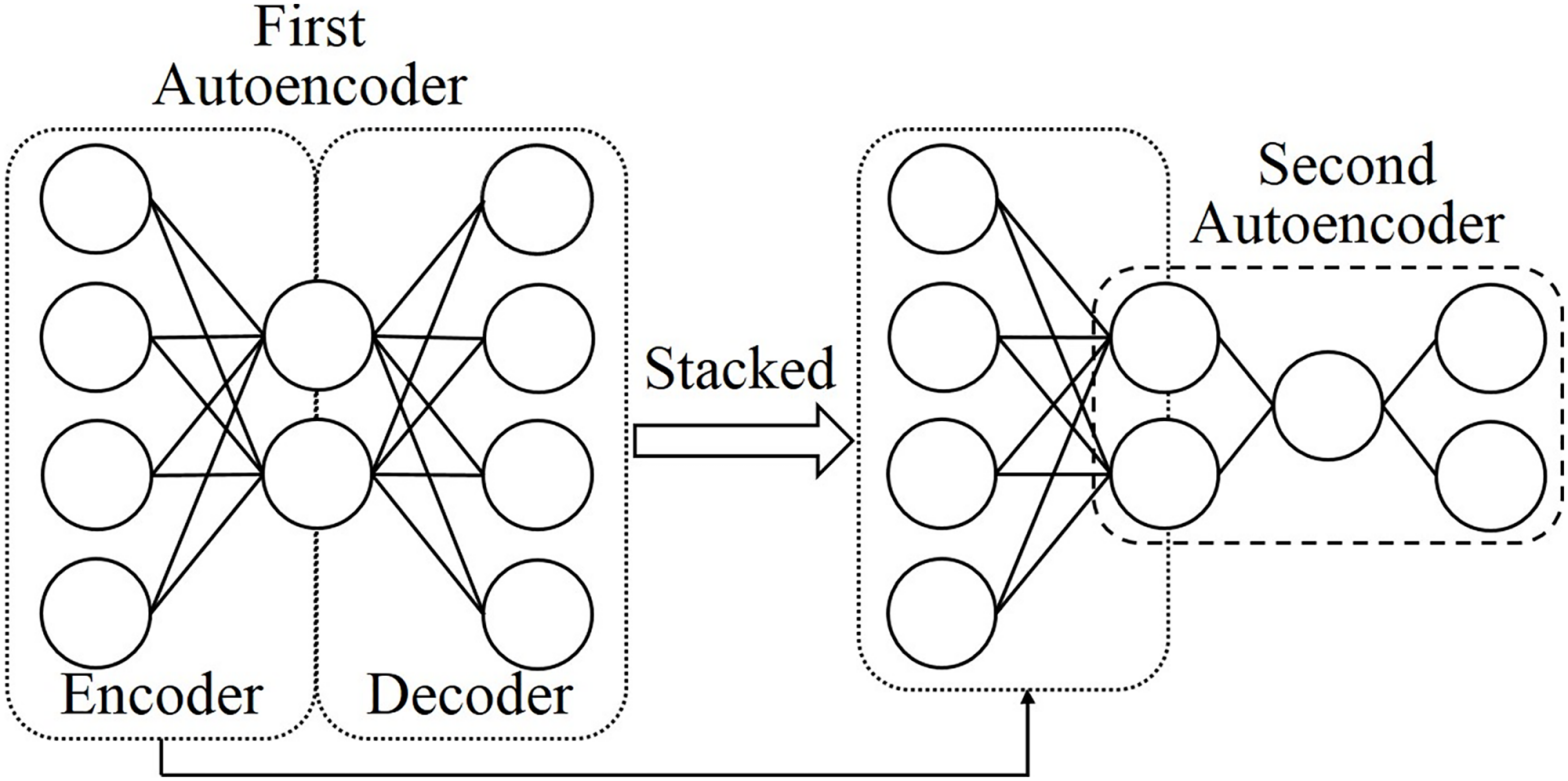
Stacked autoencoder.

Fig 2 shows a stacked AE composed of two AEs. The learning process of the stacked AE is as follows:

1. The first AE (left-hand side of Fig 2) is trained by the learning algorithm described in Section 2.1 so that it can reconstruct the input dataset.
2. The second AE is stacked on the hidden layer of the first AE (right-hand side of Fig 2).
3. The second AE is trained by the same learning algorithm so that it can reconstruct the value encoded by the first AE.

After the learning process, the stacked AE is able to reduce the dimension of the input datasets and can be used as a feature extractor.

### Tied weight

*Tied Weight* is a technique for restricting neural networks [42]; this is often used in AEs. The technique shares synapses between layers; namely, it shares synapses between the input and hidden layers, and the hidden and output layers. The weight matrix between the hidden and output layers is represented as a transposed matrix of the input and hidden layers; this is shown in Eq (10).

$$\begin{array}{c} \vec{W^{\prime }}={\vec{W}}^{T} \end{array}$$ (10)

By using the tied weight technique, AEs can be used to reduce the parameters that need to be updated and can improve the regularization ability.

## Shared synapse architecture

**Fig 3:**
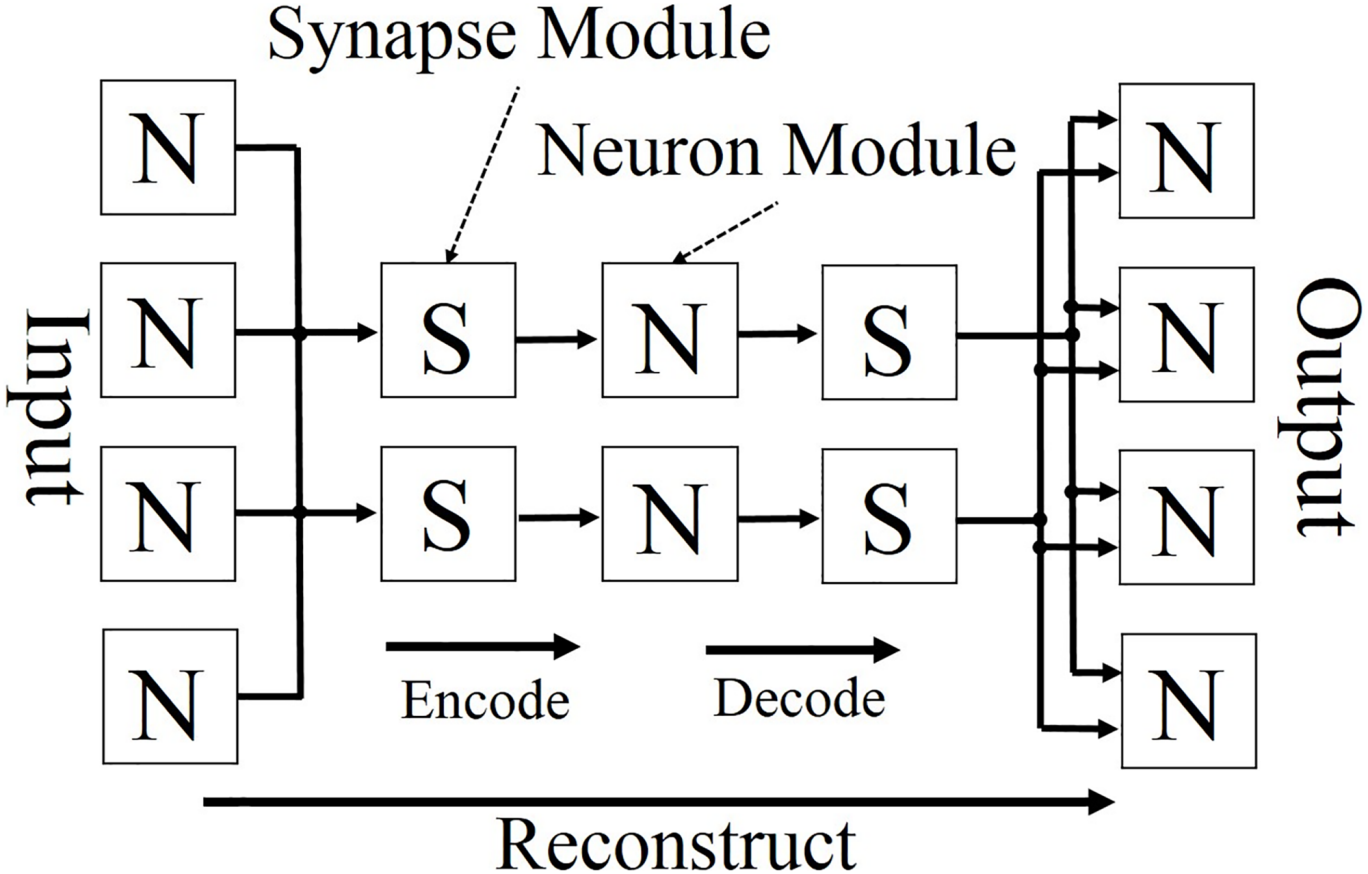
Reconstruction of AE. Reprinted from [41] under a CC BY license, with permission from Springer Nature, original copyright 2016 (S1 File).

**Fig 4:**
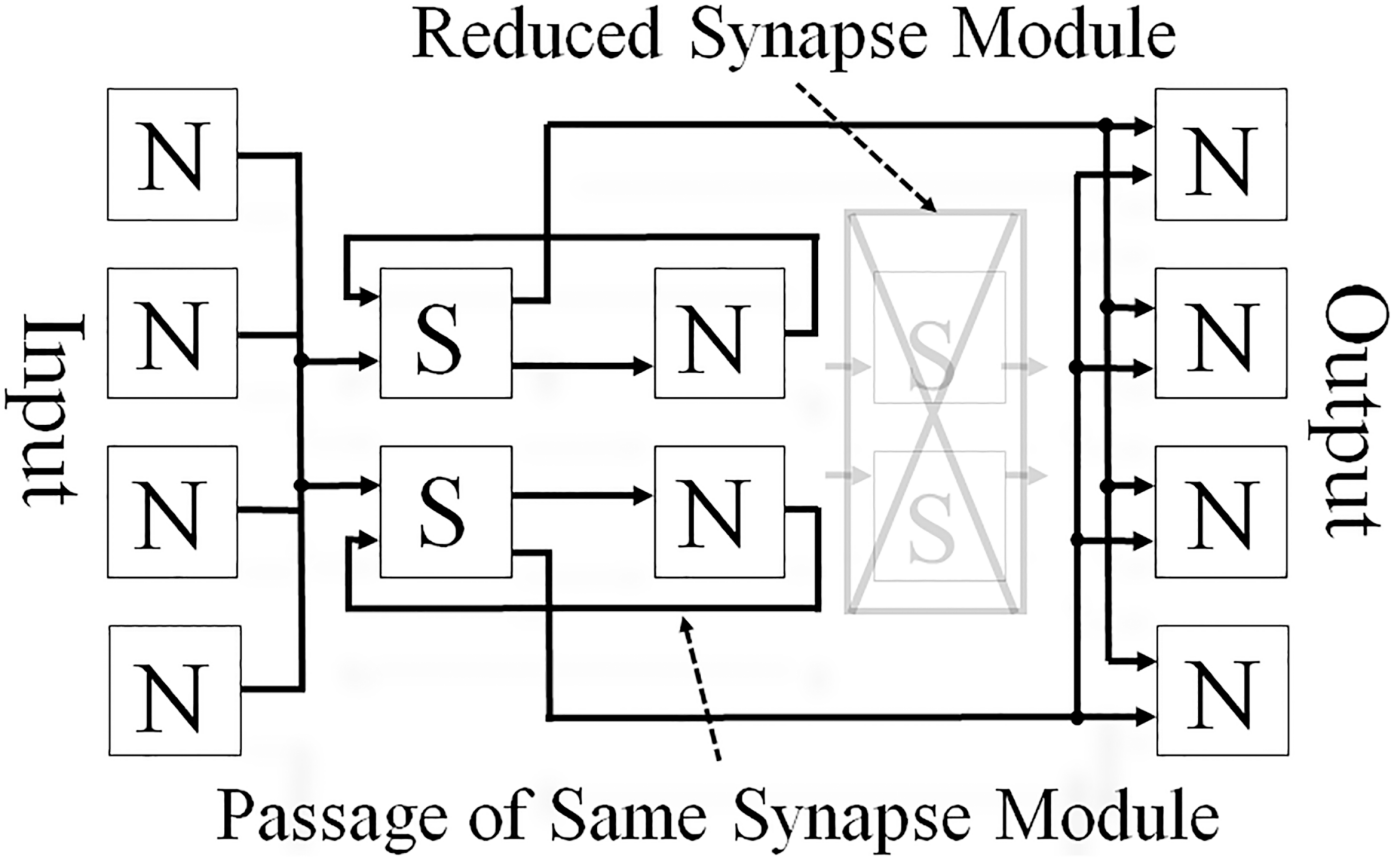
Reconstruction of AE with shared synapse architecture. Reprinted from [41] under a CC BY license, with permission from Springer Nature, original copyright 2016 (S1 File).

This paper proposes a shared synapse architecture based on the tied weight technique; the aim of this architecture is to decrease the utilizations of the recourses of the FPGAs. It is assumed that the reconstruction process for the AEs is described in the neuron and synapse modules; this process is shown in Fig 3. By sharing the synapses, the number of synapse modules used is halved; a representation of the architecture of an AE with shared synapses is shown in Fig 4. The data flow should be noted. Even though this architecture shares the synapse module, the data flow and timing of module driving do not change the operation order, which remains the same as that of a regular AE. The outputs of hidden neurons repass again the same synapse modules that were passed once; then they do not go forward to the hidden layer but to the output layer instead. Therefore, the synapse modules are executed twice according to every set of encode and decode processes.

**Fig 5:**
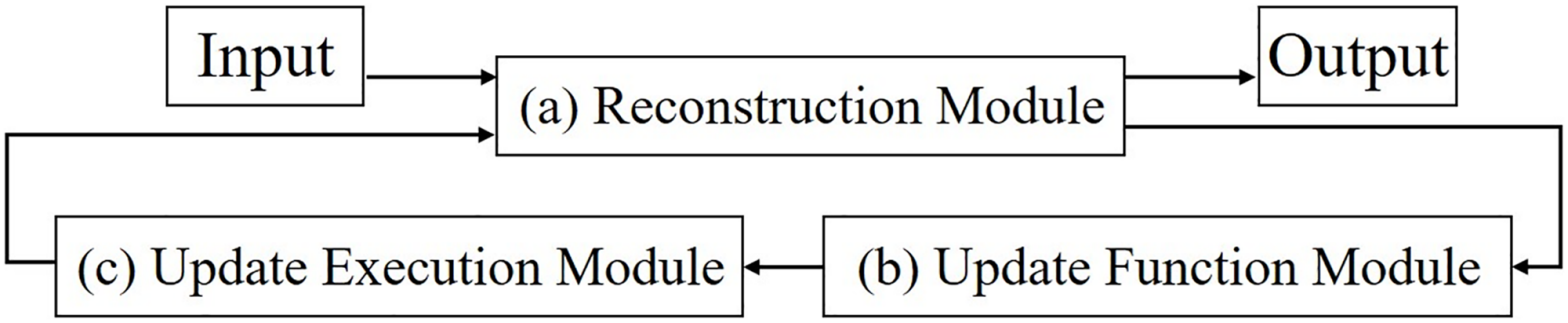
Entire circuit.

**Fig 6:**
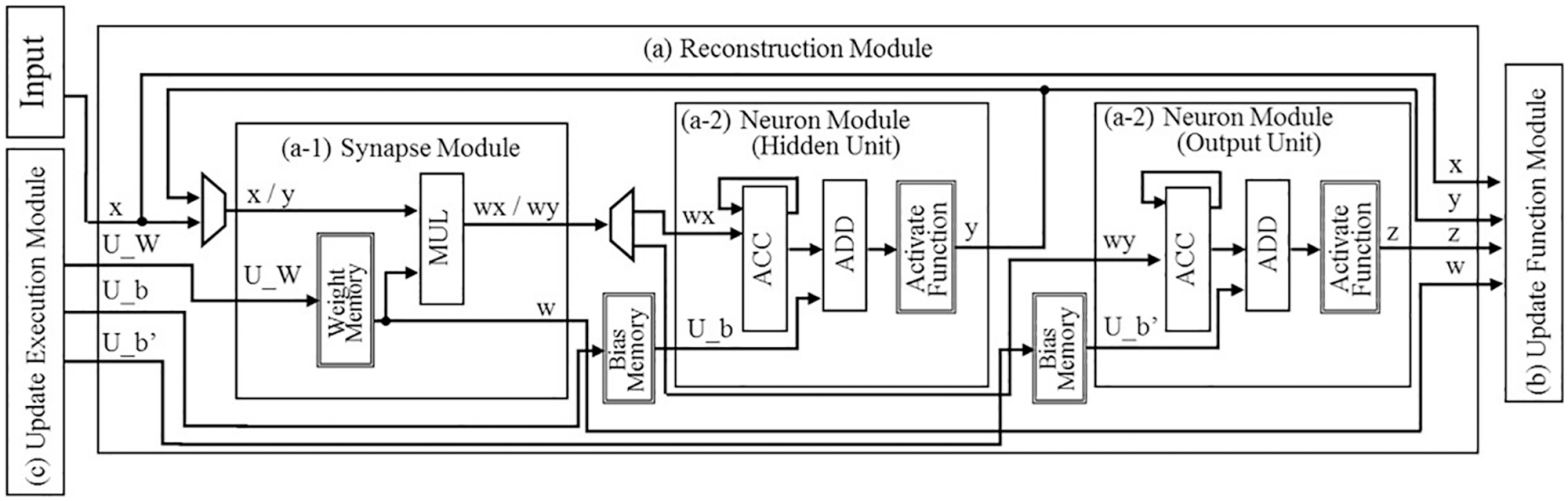
Reconstruction module.

**Fig 7:**
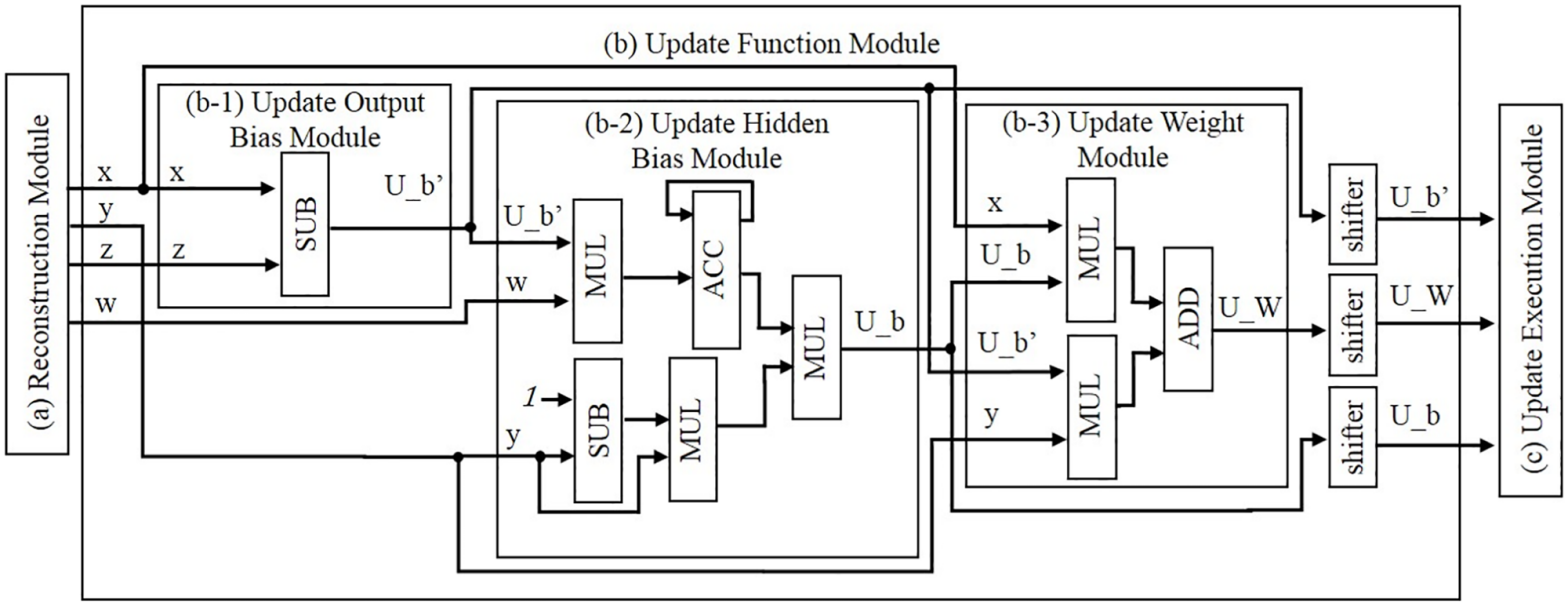
Update function module.

The circuit proposed by this study consists of three modules, as shown in Fig 5: a reconstruction module, an update function module, and an update execution module. The reconstruction and update function modules contain sub-modules, and the internal structures of each of these two modules are shown individually in Figs 6 and 7, respectively.

The roles of these three modules and their respective sub-modules are as described in the following sentences in more detail. Eighteen-bit fixed point numbers are used in the modules shown in Figs 6 and 7; these are the memory for the weights, the multiplier, the memory for the bias, the accumulator, the adder, and the subtractor. The activation function module shown in Fig 6 is designed using a look-up table (LUT), and it uses ten-bit numbers as inputs and eighteen-bit numbers as outputs. The values in the LUT can be rewritten so as to become other activation functions:

(a) Reconstruction Module
  This module is composed of two sub-modules, as shown in Fig 6 and it has a reconstruction mode and an update mode.
  - Reconstruction Mode
    Via two processes (encode and decode), the input data are reconstructed on as output data.
    a-1) Synapse Module
      A weight value is read from the weight memory. The input data and corresponding weight values are multiplied.
    a-2) Neuron Module
      The accumulation of all of the inputs and biases are determined. From the LUT, the corresponding value of the accumulation is given as a result output by the sigmoidal operation.
  - Update Mode
    This module receives the update values from the update execution module and updates all of the parameters in the weight memory and two bias memories.
(b) Update Function Module
  This module is composed of three sub-modules, as shown in Fig 7. Utilizing a given input, output, and all of the parameters sent by the reconstruction module, the update values of the parameters are determined.
  b-1) Update Output Bias Module
    An input and an output value are sent from the reconstruction module. The update value of $\vec{b^{\prime }}$, is determined by Eq (6).
  b-2) Update Hidden Bias Module
    The values for the calculation are sent from the other modules; a weight and a hidden value from the reconstruction module, and the output of the update output bias module. The update value of $\vec{b}$ is determined by Eq (5).
  b-3) Update Weight Module
    The values for the calculation are sent from the other modules; an input and an output value from the reconstruction module, the output of the output bias update module, and the output of the update hidden bias module. The update value of $\vec{W}$ is determined by Eq (4).
(c) Update Execution Module
  The update values of the parameters are given by the update function module. Each update value is added to a corresponding parameter by this module. Each updated value is then sent to the reconstruction module in the update process. Another function of this module is to switch the reconstruction module from the reconstruction mode to the update mode before the updated values are transferred to the reconstruction module. After the update, the update execution module switches from the update mode to the reconstruction mode.

## Software implementation of the proposed AE

Because the number of processes involved in the hardware implementation is greater than the number for the software implementation, it is advisable to first verify the hardware implementation using software implementation. A quantized AE was implemented into a piece of software coded by C++. The quantized AE is able to handle eighteen-bit fixed-point numbers. In addition, an array with 1,024 elements was used as a quantized sigmoidal function generator in the piece of software.

The number of input and output neurons was set to four, and the number of hidden neurons was set to two; in total, sixteen sets of four tuples of binary data were prepared for the learning process. Both the quantized AE and a regular AE with 32-bit floating-point numbers were trained with one set from the dataset for each learning experiment. The number of epochs was set to 1,000, and the learning rate was set to 0.0078125. The initial value of each parameter of the AEs was set to zero.

**Fig 8:**
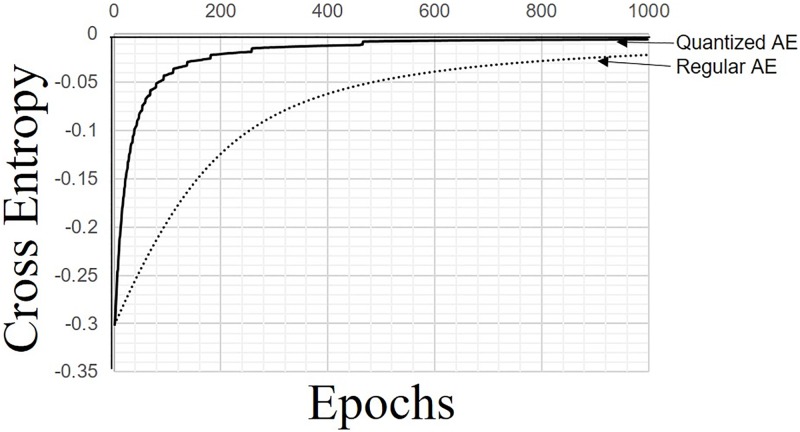
Comparison of learning results.

Fig 8 shows the averages of the results of the sixteen learning processes using the quantized and regular AEs. In the figure, the straight line indicates the results of the quantized AE, while the dotted line indicates the results of the regular AE; the vertical and horizontal axes indicate the cross entropy and epochs, respectively. The results show that the quantized AE operated better than the regular AE. The cross-entropy errors of both of the AEs decreased as the learning processes proceeded. The cross-entropy errors of the quantized AE converged faster than those of the regular AE; this was because the update process of the quantized AE operated rougher than the regular AE.

## Hardware implementation of the proposed AE

As the effectiveness of the proposed architecture was validated via the experiments conducted by the software implementation, the proposed AE was implemented in a digital circuit. As with the software implementation, the designed network had four input and output neurons and two hidden neurons. The digital circuit with the proposed design was described by the Verilog Hardware Description Language (HDL) with RTL.

### Logic synthesis of the proposed circuit

The designed circuit was synthesized using Xilinx ISE 14.7, and the target device used was a Xilinx Virtex-6 xc6vlx240t. The results of the synthesis are shown in Table 1.

**Table 1:** Logic synthesis results for the shared synapse AEs.

Module	Registers	LUTs	DSPs*	Freq. (*MHz*)
**Entire AE module**	**6,284 (2.08%)**	**6,198 (4.11%)**	**30 (3.91%)**	**230.654**
**Reconstruction**	**2,621 (0.87%)**	**2,442 (1.62%)**	**2 (0.26%)**	**242.777**
Synapse	270 (0.09%)	262 (0.17%)	1 (0.13%)	242.424
Neuron	102 (0.03%)	153 (0.10%)	0 (0%)	363.769
**Update function**	**6125 (2.03%)**	**6557 (4.35%)**	**28 (3.65%)**	**230.654**
Update output bias	667 (0.22%)	454 (0.30%)	0 (0%)	419.639
Update hidden bias	1139 (0.38%)	1190 (0.79%)	6 (0.78%)	242.777
Update weight	399 (0.13%)	411 (0.27%)	2 (0.26%)	242.777
**Update Execution**	**763 (0.25%)**	**7(0.004%)**	**0 (0%)**	**651.042**

* Digital signal processors

In addition to the modules shown in this table, several other sub-modules were used to control the weight memory; these were synthesized, and they consisted of a serial-parallel converter, a parallel-serial converter, and a signal selector. The proposed shared synapse architecture therefore reduced the number of the synapse modules as well as the number of the sub-modules belonging to the synapse module. Moreover, the larger the network that was constructed, the greater the reductions in the number of the module increase and the more effective the proposed architecture is for the implementation of AEs into FPGAs.

### Verification of learning performance

To verify the performance of the designed AE circuit, the proposed circuit was logically simulated by a Veritak Verilog HDL simulator. As in the software experiments, the proposed designed circuit was trained using sixteen sets of four-bit binary data. The leaning rate was set at 0.0078125, which corresponds to 2^−7^, and therefore the multiplication of the update values with this learning rate were replaced by seven-bit shifting. The number of the epochs was set to 365.

**Fig 9:**
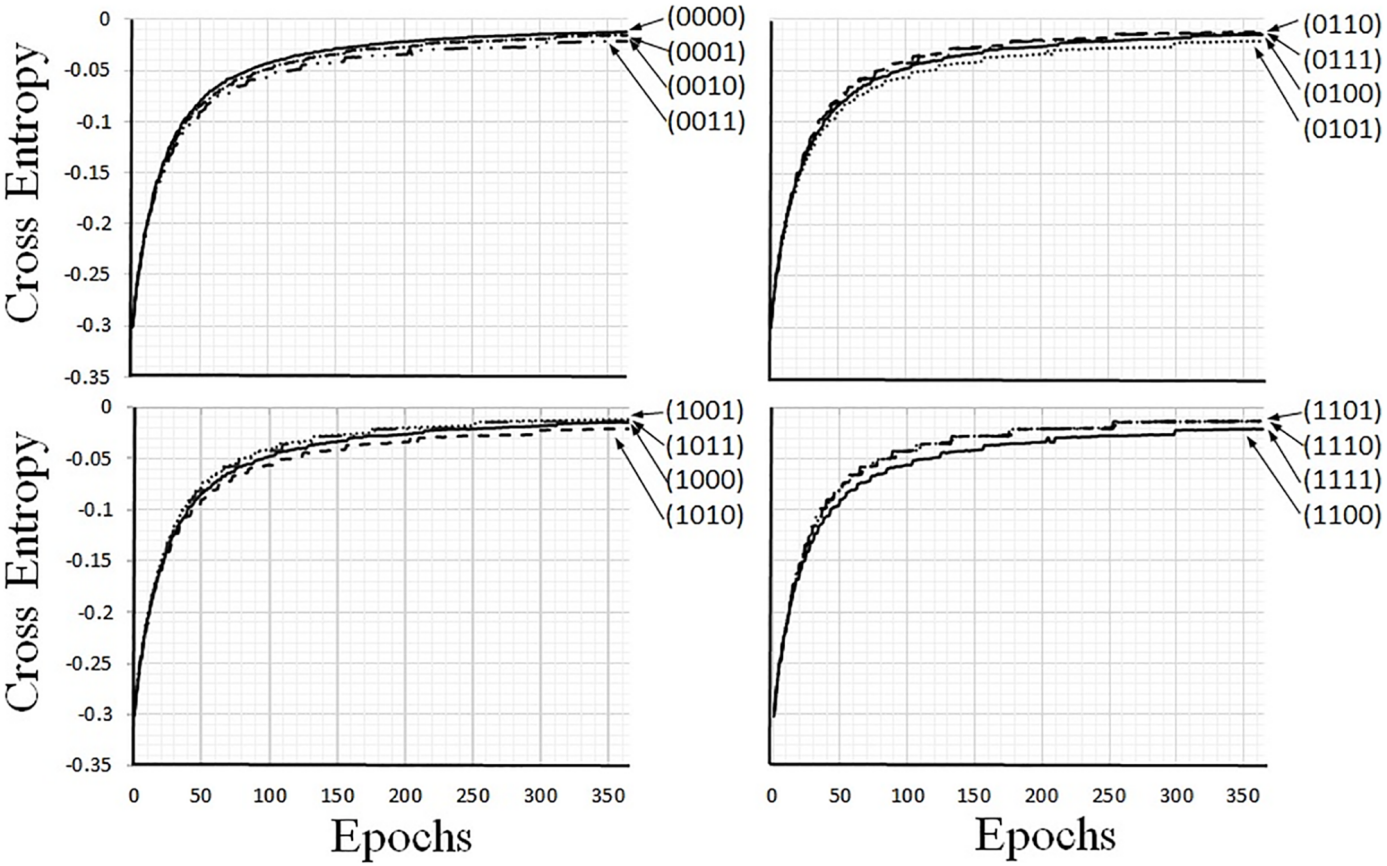
Training results represented with cross entropy errors.

The outputs produced after training are shown in Table 2. The cross entropy error between the corresponding input and output values was measured by Eq (3), and it can be seen from Table 2 that the output neurons reconstructed the corresponding input values. The averages of the four errors in each of the learning epochs are shown in Fig 9; in this figure, the vertical and horizontal axes express the cross entropy and epochs, respectively. As can be seen in Fig 9, as the number of epochs increased, the value of errors decreased. It can therefore be concluded that the AE circuit adjusted the parameters so that the input data could be reconstructed as the output data.

**Table 2:** Output results of the proposed AE circuit after training.

Input: 0 0 0 0			
0.027267456	0.027267456	0.027267456	0.027267456
Input: 0 0 0 1			
0.034179688	0.034179688	0.034179688	0.96875
Input: 0 0 1 0			
0.034179688	0.034179688	0.96875	0.034179688
Input: 0 0 1 1			
0.048828125	0.048828125	0.953125	0.953125
Input: 0 1 0 0			
0.034179688	0.96875	0.034179688	0.034179688
Input: 0 1 0 1			
0.048828125	0.953125	0.048828125	0.953125
Input: 0 1 1 0			
0.025268555	0.96875	0.96875	0.025268555
Input: 0 1 1 1			
0.025756836	0.96875	0.96875	0.96875
Input: 1 0 0 0			
0.96875	0.034179688	0.034179688	0.034179688
Input: 1 0 0 1			
0.96875	0.025268555	0.025268555	0.96875
Input: 1 0 1 0			
0.953125	0.048828125	0.953125	0.048828125
Input: 1 0 1 1			
0.96875	0.025756836	0.96875	0.96875
Input: 1 1 0 0			
0.953125	0.953125	0.048828125	0.048828125
Input: 1 1 0 1			
0.96875	0.96875	0.025756836	0.96875
Input: 1 1 1 0			
0.96875	0.96875	0.96875	0.025756836
Input: 1 1 1 1			
0.96875	0.96875	0.96875	0.96875

### Evaluation of processing speed performance

To evaluate the processing speed performance of the proposed circuits, a processing speed performance index for hardware and the number of multipliers are computed.

“Operations per second (OPS)” is generally used as a performance index for hardware processing speed, and we have employed OPS. OPS represents the number of operations per second for updating the network according to Eqs (1)–(9) during the learning phase of AEs. The processing time for updating is decided by the number of clock cycles and the operation frequency of the circuit. Details about the number of clock cycles of the proposed circuits are given in Table 3, and the number of clock cycles is computed using Eq (11) when the AE structure is 4-2-4.

$$\begin{array}{c} ClockCycles=8N^{i}+2N^{h}+235 \end{array}$$ (11)

where the number of the neurons in an input and in the hidden layers are represented by *N*^*i*^ and *N*^*h*^, respectively.

**Table 3:** Equations for the number of clock cycles.

Modules	Clock cycles
Neuron (hidden unit)	*N*^*i*^ + 5
Neuron (output unit)	*N*^*h*^ + 5
Update hidden bias	*N*^*i*^ + 17
Synapse	(*N*^*i*^ + 8) * 2
Weight memory controller	*N*^*i*^ + *N*^*h*^ + 4
Update execution	*N*^*i*^ + 8
Fixed clock cycle	164

Assuming that each addition, subtraction, and multiplication is counted as one operation, the number of operations is determined using Eqs (1)–(9), and is formulated as Eq (12). Moreover, the processing time is determined using Eq (13).

$$\begin{array}{c} \#ofOperations=6N^{i}+11N^{i}N^{h}+6N^{h} \end{array}$$ (12)

$$\begin{array}{c} ProcessingTime=\frac{ClockCycle}{Frequency} \end{array}$$ (13)

where the clock cycles and frequency are computed using Eq (11) and Table 1, respectively.

Therefore, the OPS is computed by Eq (14).

$$\begin{array}{c} OPS=\frac{\#ofOperations}{ProcessingTime}=\frac{6N^{i}+11N^{i}N^{h}+6N^{h}}{8N^{i}+2N^{h}+235}*Frequency \end{array}$$ (14)

A multiplier is synthesized as a digital signal processor (DSP), which is a specific customized primitive element of FPGAs. The number of DSPs is limited depending on FPGAs. The number of multipliers used in our circuits is determined using Eq (15) given below.

$$\begin{array}{c} \#ofMultipliers=N^{h}*\left(2*N^{i}+9\right) \end{array}$$ (15)

As expressed in Eq (15), the number of multipliers increases linearly along the equation until the limitation of DSPs.

The OPS and the number of multipliers of various structure are given in Table 4, where the 4-2-4 structure is compared with four types of structures: 8-2-8 (double the number of in-out neurons) and 4-4-4 (double the number of hidden neurons), and 20-10-20 (five times the number of both of in-out and hidden neurons), 24-10-24 (maximum structure in this setup). As mentioned above, the target device of this paper is xc6vlx240t which has 768 DSPs. DSP utilization is not limited to a multiplier and the total number of DSP utilizations is cleared by a logic synthesis. Therefore, 24-10-24 is the maximum structure and this structure employs 750 DSPs.

**Table 4:** Performance and recource comparison of various structure of AEs.

Network structure	MOPS*	Number of multipliers
4-2-4	116	30
8-2-8	201	46
4-4-4	206	60
20-10-20	1,529	470
24-10-24	1,713	550

* Mega operations per second. Bigger is better

According to Table 4, the OPS tends to be influenced by the number of synapses, which is determined by multiplexing the number of in/out and hidden neurons as expressed by Eq (15).

To construct larger AEs than the 24-10-24 structure, the proposed circuits require a memory controller and additional memory. To communicate with external modules such the memory controller and memory, the number of operations would increase, which acts as performance penalty and degrades the performance of the proposed circuits.

## Verification of the scalability of the proposed circuit

All of the modules comprising the proposed circuits have a common interface and parameters that are controlled externally. The common interface is useful for combining the modules with one another, and the parameters can be used to define the network structures for the stacked AEs.

### Common interface

**Fig 10:**
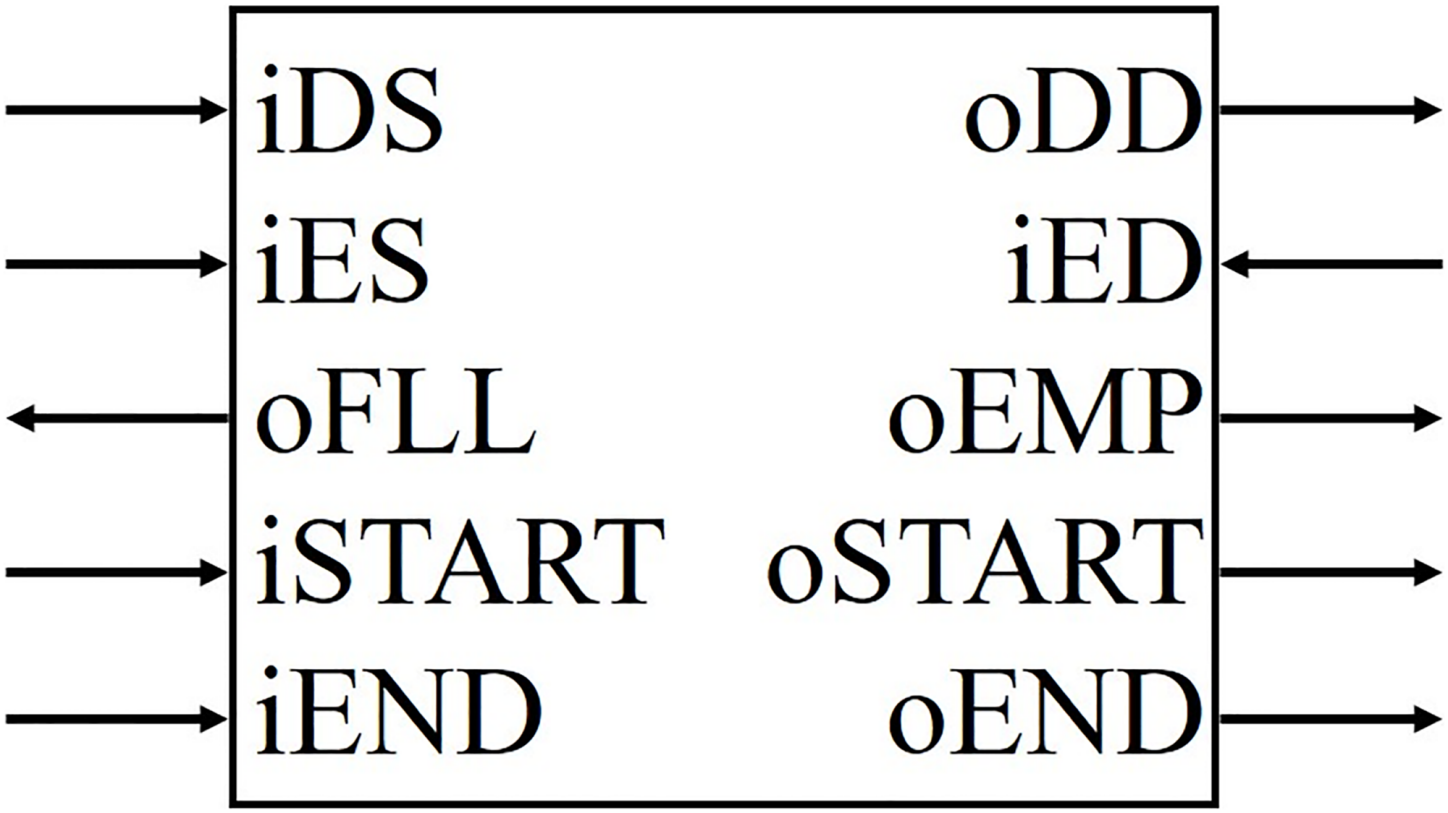
Common interface.

In our study, the common interface used was based on the first-in, first-out (FIFO) principle; this was used in all of the modules, and it was designed to help users combine the modules easily. Fig 10 shows the input and output ports: iDS and oDD are for data; iES and iED are the enable signals; oFLL and oEMP mean that the module memory is full and empty, respectively; iSTART and iEND receive the flags to start and end a process, respectively; and oSTART and oEND send the corresponding flags for starting and ending a process, respectively. A module may have several input-output signal wires for data, as iDS and oDD, depending on its function. The input and output ports of all of the modules are shown in Table 5. For instance, in Fig 6, “x”, “U_W”, “U_b” or “U_b”, (left-hand side of Reconstruction module) are regarded as iDS, and “x”, “y”, “z”, or “w”. (right-hand side of Reconstruction module) are regarded as oDD, in Fig 10 respectively.

**Table 5:** I/O ports.

Reconstruction circuit			Update function circuit		
Module	iDS	oDD	Module	iDS	oDD
(a)	x	x	(b)	x	U_b’
	U_W	y		y	U_W
	U_b	z		z	U_b
	U_b’	w		w	
(a-1)	x / y	wx / wy	(b-1)	x	U_b’
	U_W	w		z	
(a-2)	wx / wy	y / z	(b-2)	U_b’	U_b
	U_b / U_b’			w	
				y	
			(b-3)	x	U_W
				U_b	
				U_b’	
				y	

### Parameters for the network structure

#### Construction of stacked AE

**Fig 11:**
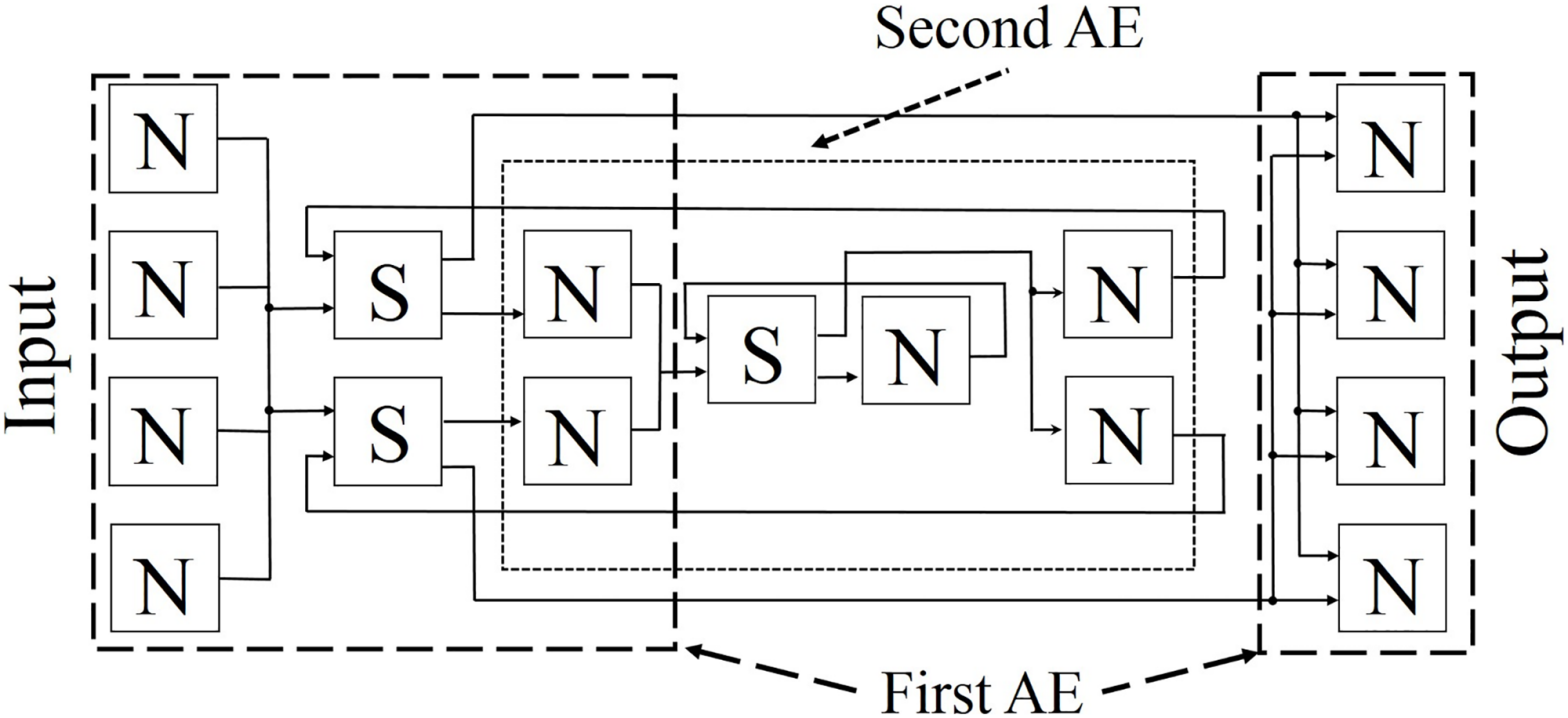
Stacked AE with shared synapse architecture.

With the parameters for the network structure in the designed circuit, we constructed the first and second AEs, which consisted of 4-2-4 and 2-1-2 networks, respectively. These two AEs were then used to construct a 4-2-1-2-4 stacked AE that had the proposed architecture; this is shown in Fig 11. As is shown in Table 6, the stacked AE circuit required fewer registers and LUTs than the first and second AE circuits did, because several of the necessary parts were duplicated when the second AE was stacked on the first.

**Table 6:** Logic synthesis results for the stacked and second AE reconstruction modules.

Module	Registers	LUTs	Freq. (*MHz*)
First AE (Reconstruction module)	2,477 (0.82%)	2,324 (1.54%)	242.424
Second AE	728 (0.24%)	798 (0.53%)	242.777
Stacked AE	2,746 (0.91%)	2,781 (1.85%)	242.777

#### Learning operation of second AE

**Fig 12:**
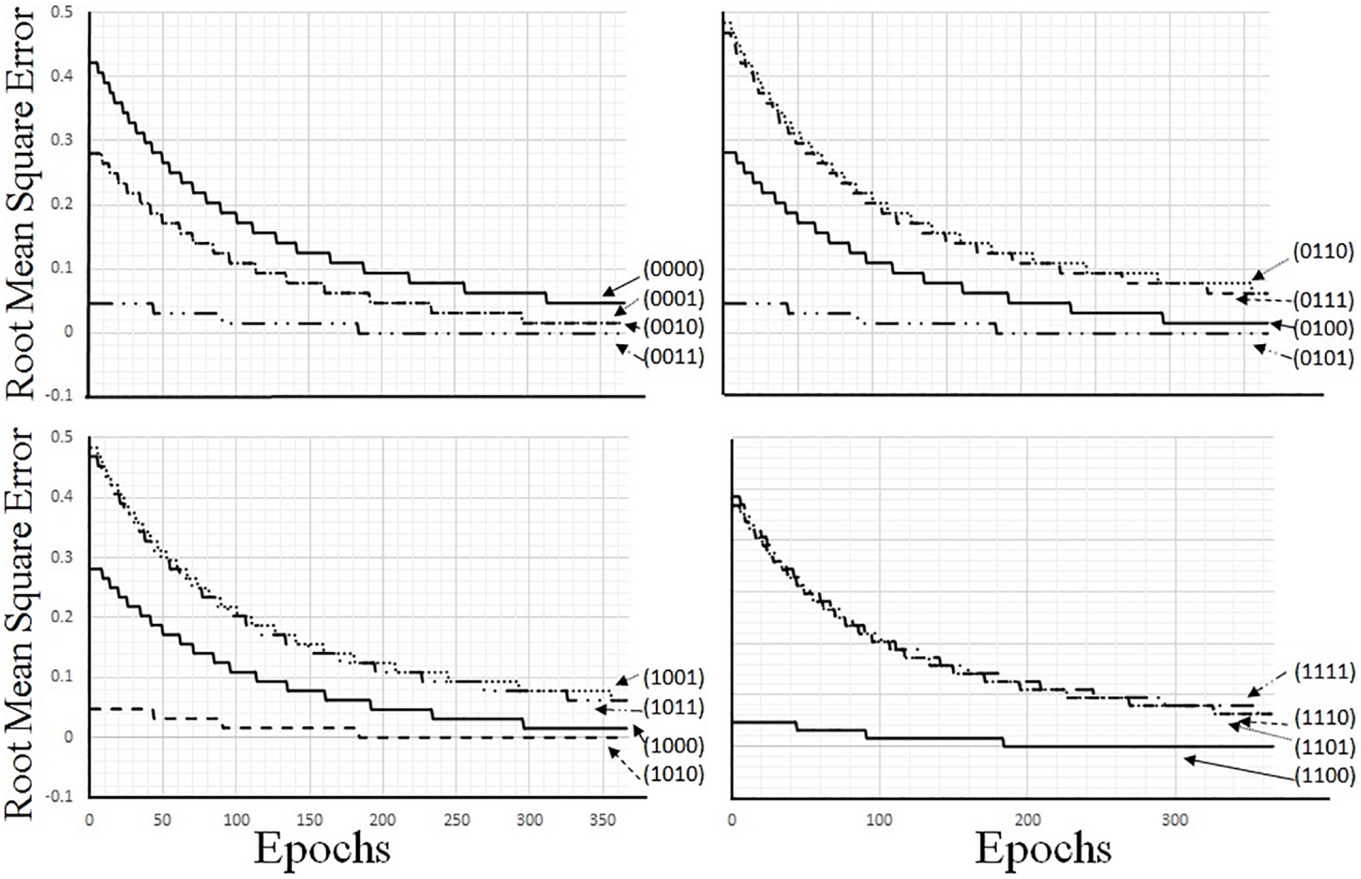
Training results represented with root mean square errors.

The learning operation for the stacked AE consisted of two steps; the first step was for the first AE, and the second step was for the second AE. Because the step for the first AE is described in Section 5, the step for the second AE is explained in this sub-section. The input data for the second AE is the value of the hidden neuron in the first AE, which had been trained with the sixteen kinds of binary data in Section 5.2. The learning rate and the number of epochs were set to the same values used in the training of the first AE. The results of the learning process are shown in Fig 12. To update the parameters of the AEs, we used Eqs (4)–(6); they were determined from the cross entropy. The root mean square error was used to measure the error in the second AE, because this error was a real value. Using the results of the experiments, we were able to confirm that all of the parameter values after training were close to the desired values.

#### Operation of the reconstruction of the stacked AE

The parameters obtained for the first and second AEs through training were input into the stacked AE. The sixteen kinds of binary datasets were used to verify whether the stacked AE was able to reconstruct the input data. The comparison between the outputs of the first AE and the reconstructed values of the stacked AE are shown in Table 7; although some of the results have an error of 20%, the output results were regarded as having successfully digitally reconstructed the input data. These errors were produced as a result of the learning process used for the second AE, i.e., the inputs were encoded into one neuron in the second AE, after they were encoded into two neurons in the first AE.

**Table 7:** Outputs of the proposed stacked AE after learning.

Input: 0 0 0 0			
0.083984375	0.083984375	0.083984375	0.083984375
Input: 0 0 0 1			
0.09765625	0.16015625	0.09765625	0.84375
Input: 0 0 1 0			
0.09765625	0.16015625	0.75	0.16015625
Input: 0 0 1 1			
0.107421875	0.203125	0.796875	0.890625
Input: 0 1 0 0			
0.16015625	0.75	0.16015625	0.09765625
Input: 0 1 0 1			
0.1484375	0.859375	0.1484375	0.859375
Input: 0 1 1 0			
0.22265625	0.796875	0.796875	0.22265625
Input: 0 1 1 1			
0.20703125	0.9375	0.8125	0.9375
Input: 1 0 0 0			
0.84375	0.09765625	0.16015625	0.09765625
Input: 1 0 0 1			
0.796875	0.22265625	0.22265625	0.796875
Input: 1 0 1 0			
0.859375	0.1484375	0.859375	0.1484375
Input: 1 0 1 1			
0.8125	0.484375	0.8125	0.9375
Input: 1 1 0 0			
0.890625	0.796875	0.203125	0.107421875
Input: 1 1 0 1			
0.9375	0.9375	0.484375	0.8125
Input: 1 1 1 0			
0.9375	0.8125	0.9375	0.20703125
Input: 1 1 1 1			
0.984375	0.984375	0.984375	0.984375

### Parameters for the bit width

**Fig 13:**
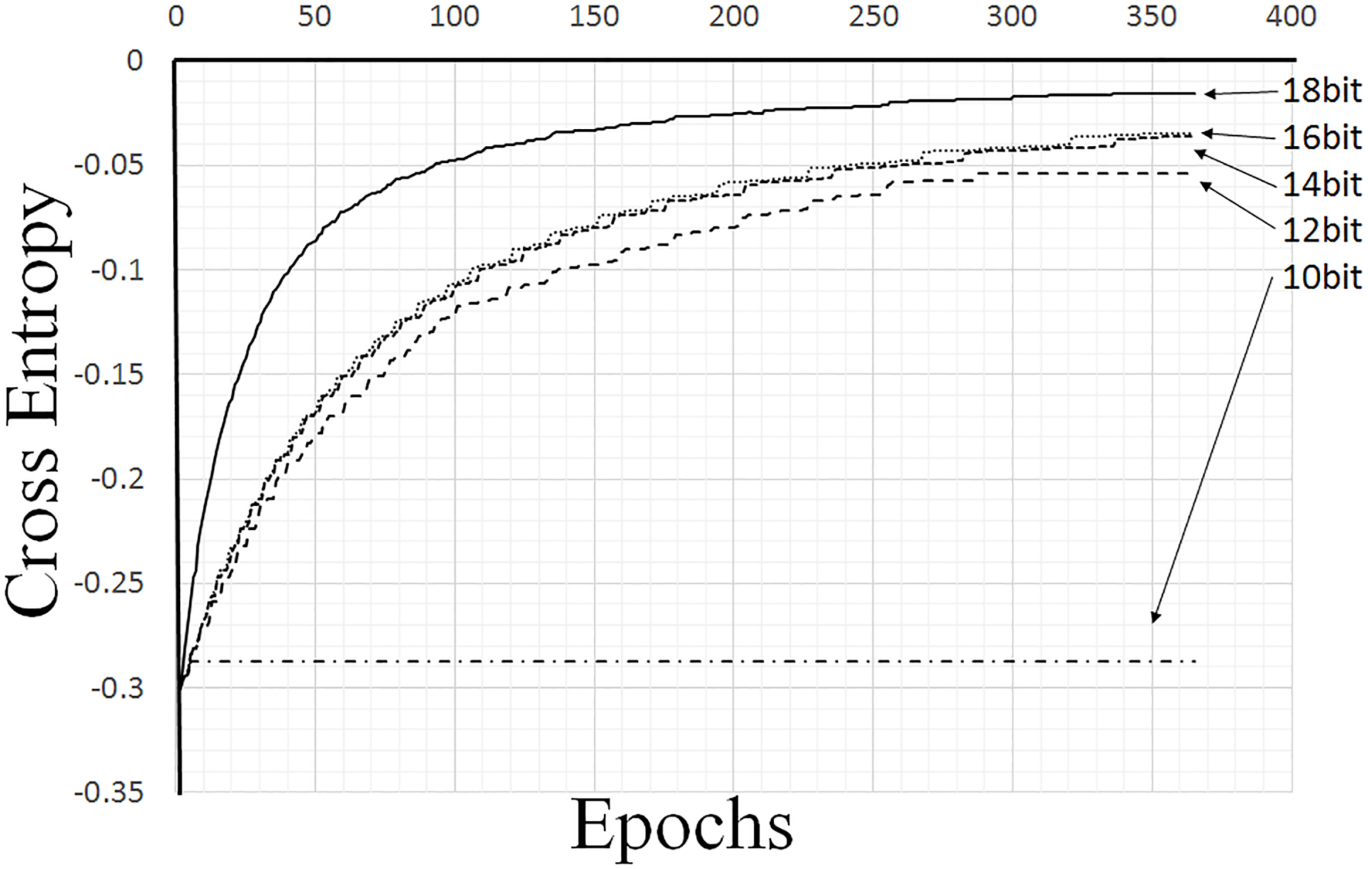
Relationship between accuracy and bit width.

To evaluate the relationship between the bit widths of the proposed circuits and the learning performances, the bit widths were changed from eighteen to ten. The results of the experiments are shown in Fig 13, and it can be seen that the AE reaches the target values more closely as the number of bits increases. In the case of ten bits, the cross entropy error was found to barely decrease; this was because seven-bit shifting, which was used for the learning rate, leads to a result that is almost zero in the update process for ten-bit numbers.

## Comparison with related works

The proposed circuit was compared with two related works about the implementations of AEs into FPGAs. The results of the comparisons are shown in Table 8.

**Table 8:** Comparison of the implementations of the AEs.

	Algorithm	Method	Logic synthesis	MOPS*
Proposed	AE (Stacked AE)	RTL	Possible	1,713
[40]	Sparse AE	Behavior	Impossible	N/A
[39]	Stacked AE	HLS	Possible	357

* Mega operations per second. Bigger is better

In one of the studies, sparse AEs are described by a Verilog HDL as a behavior model and evaluated by pre-route simulations [40]; however, there is no description in this study about the detailed hardware architecture used and the logic synthesis results. Because the pre-route behavior model does not consider FPGA resources and timing issues, implementing it in an actual FPGA is difficult. According to Table 8, the performance of [40] is not available (N/A) and cannot be calculated, because both operation frequency and time period, key requirements for evaluating the processing speed of a digital circuit, are not given in [40].

In the other study, a three-layered stacked AE was designed using an OpenCL programming framework and implemented on an NVIDIA GTX Titan GPU, a Qualcomm Adreno 330 mobile GPU, and an Altera Stratix V D5 FPGA [39]. For the FPGA implementation, HLS was used to generate an RTL code automatically from the OpenCL description. The experimental results showed that the FPGA performed the worst in terms of processing speed. Generally, it is hard to find an optimal digital architecture from a high-level description by architecture exploration in HLS. The generated RTL code was therefore not optimized for the stacked AE, and its FPGA implementation resulted in the worst performance among the devices.

In contrast, the circuit proposed in the present study was manually designed by RTL and was based on the shared synapse architecture. The RTL design enabled us to formulate the number of clock cycles required for an AE used in the proposed circuit; this is shown in more detail in Table 3. A weight memory controller controls the read/write operations of the weight memory in the synapse module; the other modules are shown in Figs 6 and 7. By considering the equations in Table 3 and the operating frequency from the synthesized results shown in Tables 1 and 6, we can estimate the theoretical performance of a circuit by constructing arbitrary networks. From these estimates, we can see that the proposed circuit designed by RTL is much faster than a circuit designed by HLS. As shown in Table 8, unlike [40], the OPS can be calculated because both the processing time for one epoch and the number of operations are given in descriptions in [39]. Compared to [39], the performance of the proposed architecture is six times better than it.

## Conclusion

A novel hardware architecture for AEs, which we have called a shared synapse architecture, was proposed in this paper. This proposed architecture meant that the number of synapse modules in the AE circuit that were implemented in could be halved. To construct various types of AEs, the proposed architecture had three parameters; one to change a network structure, another to change the bit width of the internal values of the AE, and the other to change a learning rate. Additionally, the proposed circuits had a common interface that allows them to be combined easily. We designed two AEs that consisted of 4-2-4 and 2-1-2 networks, and we combined them to construct a 4-2-1-2-4 stacked AE. The designed circuits were logically synthesized and subsequently evaluated. Our experimental results show that the proposed AE and the stacked AE circuits successfully reconstructed input data. By comparing our results with related works, we were able to discuss the effectiveness of the proposed RTL design.

Future work involves designing a memory controller that can divide AE processes along time series. A time series processes of large AE networks can be constructed using multiplexing the proposed architecture in time and implemented by the memory controller and a block RAM (high-capacity FPGA internal memory) or an external memory (SDRAM) that stores weights memories. With this future work, we intend to extend the proposed circuit to deep-stacked AEs and integrate it into embedded systems. We plan to apply an extended version of the proposed circuits to various applications such as binary value applications, image processing applications, and abnormal detection applications.

## Supporting information

S1 File RightsLink printable license. Permission from the original copyright holder of Figs 1, 3 and 4.
(PDF)
